# Astrocytic expression of HIV-1 viral protein R in the hippocampus causes chromatolysis, synaptic loss and memory impairment

**DOI:** 10.1186/1742-2094-11-53

**Published:** 2014-03-22

**Authors:** Lilith Torres, Richard J Noel

**Affiliations:** 1 Department of Biochemistry, Ponce School of Medicine and Health Sciences, P.O Box 7004, 00731 Ponce, PR, USA

**Keywords:** astrocyte, synaptophysin, learning, HAND, rat model, object recognition

## Abstract

**Background:**

HIV-infected individuals are at an increased risk of developing neurological abnormalities. HIV induces neurotoxicity by host cellular factors and individual viral proteins. Some of these proteins including viral protein R (Vpr) promote immune activation and neuronal damage. Vpr is known to contribute to cell death of cultured rat hippocampal neurons and suppresses axonal growth. Behavioral studies are limited and suggest hyperactivity in the presence of Vpr. Thus Vpr may play a role in hippocampal loss of function. The purpose of this study is to determine the ability of HIV-1 Vpr production by astrocytes in the hippocampus to cause neurological deficits and memory impairments.

**Methods:**

We tested the performance of rats in novel object and novel location tasks after hippocampal infusion with astrocytes expressing HIV-1 Vpr. Synaptic injury and morphological changes were measured by synaptophysin immunoreactivity and Nissl staining.

**Results:**

Vpr-infused rats showed impaired novel location and novel object recognition compared with control rats expressing green fluorescent protein (GFP). This impairment was correlated with a significant decrease in synaptophysin immunoreactivity in the hippocampal CA3 region, suggesting synaptic injury in HIV-1 Vpr-treated animals. In addition, Nissl staining showed morphological changes indicative of neuronal chromatolysis in the Vpr group. The Vpr-induced neuronal damage and synaptic loss suggest that neuronal dysfunction caused the spatial and recognition memory deficits found in the Vpr-infused animals.

**Conclusions:**

In this study, we demonstrate that HIV-1 Vpr produced by astrocytes in the hippocampus impairs hippocampal-dependent learning. The data suggest Vpr is a neurotoxin with the potential to cause learning impairment in HIV-1 infected individuals even under conditions of limited viral replication.

## Introduction

The impact of HIV-1 has been moderated by the use of combination antiretroviral therapy (cART) that converts the infection from a once certain death sentence to a serious, but treatable chronic illness [1,2]. Even so, significant neuronal dysfunction has been reported in the CNS [3,4] in over 50% of those infected even when viral loads are low to undetectable [5,6]. The mechanisms that mediate ongoing neurocognitive impairments in patients on suppressive cART remain an active area of study [7,8].

One of the major impediments to developing therapies that specifically target HIV-associated neurocognitive disorders (HAND) is the incomplete understanding of the mechanisms that are responsible for the development and progression of neurological impairments associated with HIV-1 infection. Macrophages and microglia represent the major productively infected cell in the brain, while astrocytes are considered to harbor a predominantly latent infection [9-14]. Particularly in patients on suppressive cART with undetectable plasma and cerebrospinal fluid (CSF) viremia, even low levels of viral activity in astrocytes may provide a source for viral neurotoxins. The viral burden in astrocytes, which harbor proviral DNA even during early asymptomatic infection [15], has been linked to the severity of HAND [16]. Although astrocytic infection is best characterized by limited production of early HIV-1 gene products [17-19], there are also reports supporting very low levels of replication after initial infection or by reduction of a number of host cell restriction factors [12,20-22]. Thus, in a patient on suppressive cART where replication in microglia is controlled, astrocytes are likely to have an important role in HIV neuropathology.

Astrocytes are the most abundant cell type in the brain, and they play multiple roles in brain development and normal brain functions including maintenance and regulation of neuronal cells. Evidence confirms their roles modulating neuronal signal transmission and synaptic plasticity [23-25]. Brain function may result from the combined network activity between neurons and glia. Changes in any of these interactions by HIV infection could severely impair brain homeostasis and contribute to viral neuropathology [26]. Viral replication, even at a low level, results in production of excess of viral proteins, many of which are neurotoxic. HIV-1 Vpr is a conserved HIV accessory protein involved in G2 cell cycle arrest [27] and apoptosis in human neuronal cells [28,29]. Vpr contributes to cell death of cultured rat hippocampal neurons [30], suppresses axonal growth [31] and causes increased intracellular calcium and plasma membrane permeability in neurons [32]. It is detected in the CSF of HIV patients [33,34]. Vpr has been found in the cytoplasm of astrocytes near areas of inflammation in HIV encephalitis indicating the potential for *in vivo* effects on astrocytes as well [35]. In astrocyte cultures, Vpr induces several inflammatory gene products including CCL5 [36], IL-6, IL-8, MCP-1, MIF [37], and the cyclin dependent kinase inhibitor p21/WAF1 [38]. In contrast to cellular studies of effects on astroctyes and neurons, few studies have examined whether Vpr is sufficiently neurotoxic to cause learning impairments. Vpr expression from brain monocytes in a transgenic mouse showed synaptic injury and disruption of neurotransmitter homeostatic enzymes as well as hyperexcitability and aberrant motor activity [28]. While these findings indicate that Vpr can alter CNS function, it is unknown whether Vpr expressed by astrocytes contributes to neurocognitive impairments. In this study, we established a model system to test whether Vpr produced by astrocytes can induce sufficient neurotoxicity to manifest as impaired learning. The purpose of this study is to determine the ability of HIV-1 Vpr production by astrocytes in the hippocampus to cause neurological deficits and memory impairments.

## Materials and methods

### Animals

All protocols involving rats were evaluated and approved by the Institutional Animal Care and Use Committee (IACUC). Sixty day old male Sprague Dawley rats obtained from the Ponce School of Medicine Animal Research Facilities were surgically implanted with autologous, Vpr-transfected astrocytes. Primary cultures of rat astrocytes were harvested from an additional rat group, also approved by the IACUC. To deliver the Vpr-transfected (or control green fluorescent protein (GFP)-transfected) cells, we used a stereotaxic device and an infusion pump system. The infusion rate was 0.5 μL/min to deliver a cell content of 100,000 cells. Prior to surgery, rats were anesthetized with isoflurane. Unilateral micro-infusion of transfected primary astrocytes was performed in the dentate gyrus of the right brain hemisphere. The infusions were at the specified coordinates: AP -0.28 mm, ML ±0.10 mm and DV -0.40 mm from bregma. Infusions were performed using a published protocol [39,40] with modifications [41]. After two days of recovery, rats were tested in the novel location and novel object recognition learning task (details follow). Following behavioral testing, rats were euthanized with an overdose of pentobarbital, blood was removed by cardiac puncture and animals were perfused with saline followed by buffered formalin to preserve brain tissue for analyses. The proper location of the infusion site was determined histologically. Throughout the experiments, rats were maintained on a 12-hour day/night cycle with free access to food and water.

### Transient transfection of primary astrocytes

Primary astrocytes were isolated from Sprague Dawley rats [41,42] and cultured in DMEM with 10% fetal bovine serum. Transfection was used as a way to deliver the plasmid-encoding viral protein Vpr to stimulate the cell protein production. Primary rat astrocytes were transfected with a plasmid-encoding Vpr-GFP (experimental group) and GFP (control). Transfections were done using the Gene Pulser Xcell Electroporation System (Bio-Rad, Hercules, CA, USA) under the following conditions: 4 mm cuvette containing 5 ug of plasmid DNA per 1.6 × 10^6^ cells in a 300 μl volume of serum-free RPMI; 250 V and 35 ms using a time constant protocol. Flow cytometry was done using a GFP plasmid to determine transfection efficiency. Routine transfection efficiency by GFP expression was between 60 and 80% positive cells.

### Vpr mRNA and protein detection

Total RNA and protein were isolated from transfected cells after various times in culture using Nucleospin reagents and protocol (Macherey-Nagel, Bethlehem, PA, USA). RNA (1 μg) was converted to cDNA using the Bio-Rad iScript cDNA synthesis kit, followed by 35 cycles of real-time PCR with the Bio-Rad SYBR-green Supermix. The GFP control transfections produced only background fluorescence values, precluding a fold-change comparison; thus, relative expression of Vpr was determined by comparing signal strength versus the housekeeping gene, β-actin, and the difference in cycle of detection (ΔCt) is reported. Western blotting was used to confirm Vpr protein expression; 25 μg of protein from each total cell lysate were separated by SDS-PAGE and transferred to PVDF membranes. Membranes were incubated overnight at 4°C with primary antibody rabbit anti-Vpr (AIDS Research and Reference Reagent Program, NIH; Cat. #11836; 1:500) or anti-actin (Sigma, St. Louis, MO, USA; Cat. #A5060; 1:1000) Detection of bound primary antibodies was achieved with horseradish peroxidase (HRP)-conjugated secondary antibody followed by chemiluminescent/chemifluorescent detection (General Electric RJP 2332).

### Novel location and novel object learning task

Novel location and object recognition tasks were performed after infusion of astrocytes expressing HIV-1 Vpr or GFP. The protocol was adjusted from Benice and colleagues [43]. These experiments start by handling the rats to provide a period for adjustment to manipulation by humans and to reduce stress that could interfere with the behavioral test. Rats were handled for 5 minutes daily over four days, prior to brain surgery (day 5). After brain surgery and a 2-day recovery period, rats began training and testing. Training consisted of a day of habituation where the rats are familiarized with the open field that is used for testing. Rats were placed in a square box (9 sq. ft. area) for two trials of 5 minutes, each separated by 1 hour. On the following day, three objects were placed at specific locations within the same open field. Rats were then trained to learn the object locations through three 5-minute exposure periods, separated by 1-hour intervals. After 24 hours, one object was moved to the opposite corner (new location) and then the rat was given a 5-minute challenge to test memory of the old location. Memory was indicated by the rat spending additional time exploring the new location as rats will preferentially explore novelty when presented with a familiar environment. One hour later, one of the two unmoved objects was replaced (new object). The rat was given a 5 -minute challenge to assess memory of old objects. The test measured exploration time for all objects, with learning indicated by a preference for the new object. Animals that did not show preference for novelty in either test were considered to have a memory deficit. Data acquisition was by a digital video camera followed by analysis with Ethovision software (Noldus Information Technology Inc., Leesburg, VA, USA).

### Nissl staining for assessment of chromatolysis and neuronal damage

After sacrifice, brains were removed and preserved in buffered formalin prior to paraffin embedding. Brain slices of 4 μm thickness were dewaxed in xylene substitute followed by rehydration with graded ethanol from 100% to 50%. Slides were incubated in distilled water, stained in thionin solution, rinsed and incubated in 50% ethanol. Slides were placed in a solution of 70% ethanol and 95% acetic acid. Final incubations were in 95% ethanol and twice in 100% ethanol solution. The slides were dipped in xylene twice. Then permount and a cover slide were added. Photos were taken using a digital camera and an Olympus microscope; images were analyzed using NIS Elements software.

### Immunohistochemistry for assessment of synaptic injury

To examine changes in synaptophysin between control and HIV-1 Vpr exposed rats, tissue sections from each group were processed for immunocytochemistry. The samples were cut at 4 μm thickness with a microtome (Microm HM340, Microm International) and fixed to positively charged microscope slides. Fixed tissues were deparaffinized in xylene substitute for 30 minutes, rehydrated through graded alcohols and neutralized with 3% hydrogen peroxide (Sigma-Aldrich), followed by a rinse under running tap water and immersion in antigen-retrieval solution (0.01 M citrate, pH 6.0) for 1 minute at 98°C. Then sections were washed in TBS for 5 minutes and treated with blocking solution containing normal goat serum (BioGenex, cat# HK112-9KE). Sections were incubated for 24 hrs at 4°C in mouse monoclonal anti-synaptophysin antibody (Neuromics, cat # MO20000, 1:500 dilutions). Negative controls with TBS instead of primary antibody were run in each slide. Primary antibody was washed in TBS buffer for 2 × 5 minutes and incubated with Multi Link secondary antibody (Super Sensitive Link-Label IHC Detection System, cat# LP000-ULE, BioGenex, San Ramon, CA, USA). Secondary antibody was washed in TBS and incubated in ABC-HRP, washed in TBS buffer and incubated in 3,3′-diaminobenzidine (cat# HK153-5KE, Biogenex, San Ramon, CA, USA). Slides were rinsed in water and counterstained with hematoxylin for 30 sec. The sections were rinsed, dehydrated and mounted with Cytoseal XYL (cat# 8312-4, Richard Allan Scientific, Kalamazoo, MI, USA). For quantitative densitometry, images of regions of interest (ROI) in the CA3 were captured from 5 rats in each group using NIH Image J 1.50 software.

### Statistical analysis

Statistical analyses were done with Graph Pad Prism version 5.02. ANOVA was used to measure statistics of locomotor activity, center zone time, and time spent exploring novelty within group Vpr and GFP, as well as analyses of Vpr expression and of differences in chromatolysis and synaptophysin. Statistical comparisons of time spent exploring new location/object between Vpr and GFP were done using the Student *t* test. *P* values ≤0.05 were considered significant.

## Results

**Figure 1:**
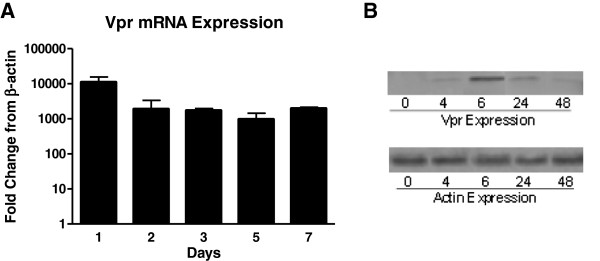
**HIV-1 viral protein R (Vpr) is expressed at mRNA and protein levels by primary astrocytes. (A)** Strong Vpr mRNA expression is maintained in culture over 7 days as determined by real time RT-PCR. Relative expression versus controls lacking Vpr are presented. β-actin was used as the normalization control. **(B)** Vpr protein peaks at 6 hours after transfection and remains detectable for 48 hours in culture as determined by western blotting.

As a preliminary step in generating a rat model of spatial learning impairment by endogenous expression of HIV-1 Vpr protein from astrocytes, we established a primary astrocyte cell culture system. Cells were cultured *in vitro* as previously described [41] and genetically modified to express Vpr from a plasmid by transfection. Figure 1A demonstrates that primary rat astrocytes strongly express the Vpr gene for at least 7 days post-transfection *in vitro* compared to control cells that were mock transfected. The mRNA levels for Vpr were substantial at several thousand-fold over background at all time points as assessed by real-time RT-PCR. A no-RT control showed background levels equivalent to the control cells (not shown) indicating the signal was from RNA and not from transfected plasmid. Vpr protein expression *in vitro* peaked rapidly and declined after 48 hours. The peak expression as determined by western blot occurs immediately after transfection at 6 hours (Figure 1B). Thus, while mRNA levels were continuously expressed, protein was detectable for a shorter duration; consistent with this *in vitro* finding, we were unable to detect Vpr protein at the time of sacrifice (data not shown). Difficulty in detecting Vpr protein *in vivo* has been reported [28] even when the toxic effects of Vpr are present.

### Normal locomotor, anxiety and weight changes during learning session

**Figure 2:**
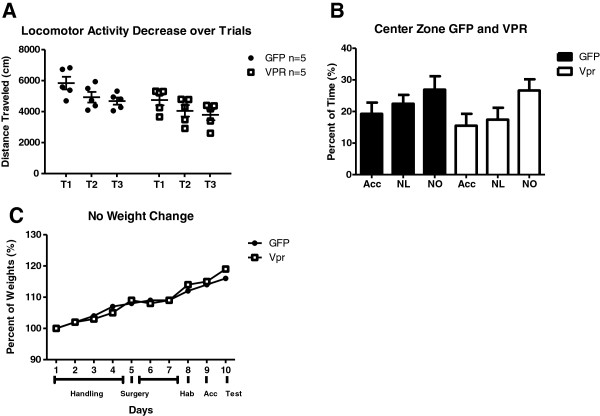
**Normal locomotor, anxiety and weight changes during learning session. (A)** Decreased distance traveled across trials shows that both groups successfully acclimated and were familiar with the environment. **(B)** Time spent in the center zone during the behavior was not altered by treatment, showing that viral protein R (Vpr) did not affect anxiety. **(C)** Normal rat weight (grams) gain shows no difference throughout the experiment or by treatment. Acc, acclimation; NL, novel location; NO, novel object.

To define any physical or behavioral interference during the learning task, we analyzed the distance traveled (locomotor function) and the percent of time the rats spent in the center (assessment of anxiety) in an open field test. In order to examine spatial learning, the first step was to familiarize the rats with the environment. Exposure to a new environment leads to habituation characterized by a large amount of initial exploratory activity and a subsequent decrease as the rats become familiar with the environment [44,45]. In our experimental design, the initial exposure to objects in the open field was where this type of habituation during learning occurred. We measured distance traveled during the trials of the acclimation phase. Animals in both groups showed a similar trend of reduction in exploration across subsequent trials (Figure 2A). While Vpr animals traveled less distance in each trial, they showed the same tendency of reduced exploration from trial 1 to trial 2 and trial 2 to trial 3 as the GFP group. This demonstrates that the Vpr rats habituated and were able to familiarize with the objects and learning environment. The groups did not show differences in anxiety as indicated by the increased time spent passing through the center zone of the open field with each exposure to the environment (Figure 2B). Finally, the neurotoxicity of Vpr we detected in the chromatolytic neurons did not affect the overall health of the rats as assessed by weight gain (Figure 2C). Based on these parameters, the rats from both the Vpr and GFP groups were able to adequately perform our tests of novel recognition without influence from motor, anxiety or general health impairments. Thus, we proceeded with the test of novelty recognition.

### Vpr expression in the hippocampus impairs long-term memory

**Figure 3:**
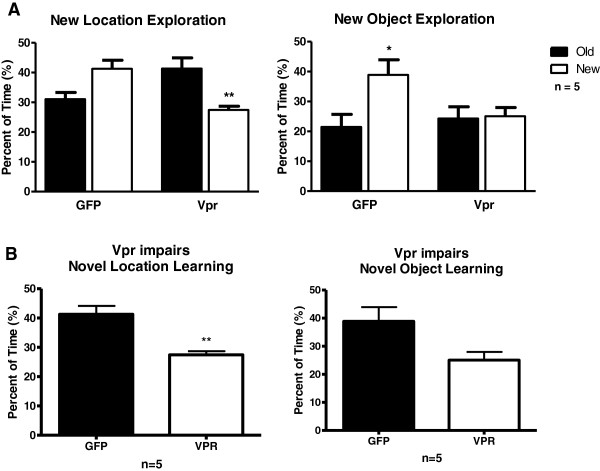
**HIV-1 viral protein R (Vpr) expression in the hippocampus causes memory impairment. (A)** Exploration time show a significant difference in learning for the control group (green fluorescent protein, GFP) and significant learning impairment for the experimental group (Vpr). Exploration time of the replacement with a novel object, show significant learning for the control group (GFP) and the familiar object show impaired learning for the experimental group (Vpr). Significance was assessed by ANOVA. *P <0.05, **P <0.01. **(B)** Significant difference in object exploration at the novel location (*t* Test *P* <0.0011). Control group (GFP) shows preference for the novel location and the novel object.

Hippocampal function (learning) can be compromised by neuronal damage or death caused by exposure to Vpr. In order to test for deficits in learning and memory due to neuron disruption driven by Vpr neurotoxicity, we measured behavioral responses to spatial change. While the rats habituate and learn the objects, a process of recognition and identification leading to prioritized attention to unexpected or new information develops [45]. This is shown when a change in spatial configuration provokes an increase in exploratory activity of the novelty. The rats exhibit a preference for the change or moved object. Spatial memory (novel location learning) and episodic memory (novel object learning) were assessed by comparing the time exploring the static object and the time exploring the moved or changed object. We determined the potential of Vpr to cause learning deficits by comparison against control animals infused with GFP-expressing astrocytes. Animals in the Vpr group showed deficits in both spatial and episodic memory by failing to increase exploration of the object in the new location and new object, respectively (Figure 3A). In contrast, GFP-treated rats displayed a trend (*P* = 0.0607) of increased exploration of the new location and statistically increased exploration of the novel object (*P* <0.05). To substantiate that the changes in exploration represented different behavior between the groups, we also made direct comparisons between the GFP- and Vpr-treated rats for their exploration of novelty. Figure 3B shows that GFP rats explored the novel location statistically more than their Vpr-treated counterparts (*P* = 0.0011). Together these results indicate a significant spatial learning impairment is caused by endogenous Vpr expression.

### Astrocyte expression of HIV-1 Vpr in the hippocampus induces structural changes indicating damage to neurons

**Figure 4:**
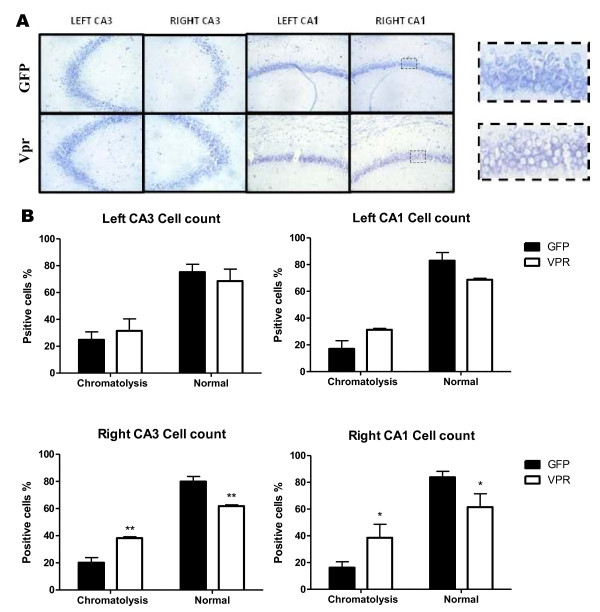
**Astrocytic HIV-1 viral protein R (Vpr) production induces neuronal chromatolysis. (A)** Representative 40× images of the Nissl stained hippocampal sections show reduced staining in Vpr-treated tissues. Chromatolysis was detected by prominent migration of the nucleus toward the periphery of the cell (shown in zoom to right). **(B)** Normal and chromatolytic neurons were counted from three sections per rat in CA1 and CA3 of both hippocampal hemispheres of green fluorescent protein (GFP)- and Vpr-treated rats. Mean and standard deviation are graphed. Significance was assessed by ANOVA. *P <0.05, **P <0.01. Five rats were counted in each group.

Astrocytes respond to viral neurotoxins by releasing proinflammatory molecules, which attract macrophages and induce apoptosis of bystander cells [46,47]. HIV Vpr is known to produce apoptosis in several types of cells including human neurons [28,29,37,46,48]. We used Nissl staining to assess neuronal morphology. Our results show that rats infused with Vpr-transfected astrocytes develop chromatolytic neuronal morphology in CA3 and CA1 (Figure 4A). We found a significant difference in number of chromatolytic cells and normal cells present in the CA1 and CA3 (Figure 4B). Chromatolysis is characterized by transient enlargement of the nucleus and cell body, reorganization of rough endoplasmic reticulum, and gradual disappearance of Nissl bodies by loss of RNA basophilic staining [49-52] Neuronal recovery through regeneration can occur after chromatolysis [53], but most often it is a precursor of cell death or apoptosis. TUNEL staining of brain slices from these rats did not show reactivity (data not shown), indicating that the chromatolysis in our model does not induce neuronal death. We also looked for activation of caspase 3/7 and Annexin V assay in astroctyes transfected with Vpr *in vitro* and did not observe signs of apoptosis (data not shown).

**Figure 5:**
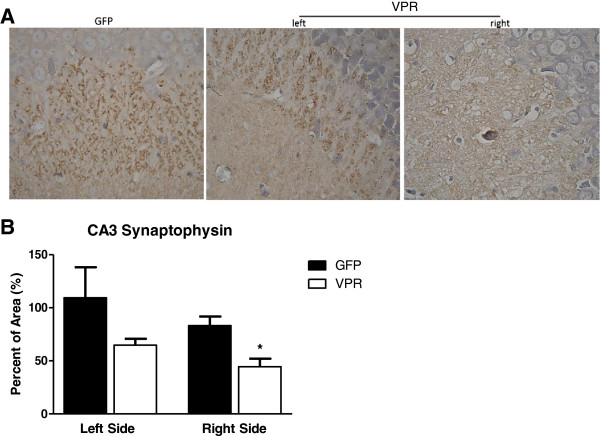
**Astrocytic HIV-1 viral protein R (Vpr) expression decreased synaptophysin immunoreactivity (A)** Representative light photomicrograph showing the distribution of synaptophysin immunoreactivity in the rat hippocampal CA3 formation. Green fluorescent protein (GFP) right side. Vpr shows both left and right. Magnification 100×. **(B)** Densitometric analysis revealed significantly decreased mean value for the Vpr group compare to control. Significance was assessed by ANOVA. *P <0.05 Graph shows mean plus standard deviation. N = 5, 3 images per rat.

Since chromatolysis may reflect damage to neurons that is below the threshold to cause apoptosis, we examined the brain tissue for signs of synaptodendritic injury. Since deletion of the major presynaptic terminal protein, synaptophysin, produces impairment in tests of object novelty and spatial learning in mice [54], we examined whether infusion of the Vpr-expressing astrocytes affected synaptophysin expression in the hippocampus. We found a significant reduction in synaptophysin staining in CA3, particularly on the infusion side (Figure 5). Thus, even though we found no evidence of neuronal death by apoptosis, the overall cellular morphology showed significant damage and increased chromatolysis that upon closer examination showed signs of synaptic injury and loss of a major synaptic protein.

## Discussion

HIV invasion into the brain promotes cellular responses causing neurological impairments that affect cognition, behavior and motor function [5,26,47,55]. In this study, we report the contributions of HIV-1 Vpr to hippocampal loss of function and neurocognitive impairment. HIV-1 Vpr produced by astrocytes in the hippocampus impairs memory and causes damage to neurons resulting in synaptic injury without detectable apoptosis. Our results support that limited viral protein production by astrocytes is sufficient to produce toxicity and functional impairment of neurons.

HIV-1 Vpr has been found in several infected cell types in the brain including astrocytes [35]. Although astrocytes show low permissiveness to HIV-1 infection [9,56,57], more recent work indicates astrocyte infection is substantially greater in HIV patients with dementia [16]. Astrocytes participate in extensive neuron-glial synaptic interactions [58] and are involved in synaptic plasticity [59,60]. Memory and synaptic plasticity have been always attributed to neuronal modulation. As reviewed by Ota [61], evidence shows that astrocytes have an ongoing role in the regulation of neuronal activity through the release of gliotransmitters and modification of postsynaptic neurons. In the hippocampus, 62% of synapses are contacted by astrocytes at the synaptic cleft [62]. Thus, even with low permissiveness, astrocyte infection could have significant effects and contribute to HAND.

The recognition that HAND occurs even when viral loads in plasma and CSF are undetectable suggests that viral activity and chronic inflammation are present during suppressive cART [5,8]. The brain serves as a reservoir for HIV and represents a site in which this residual viral activity continues [63]. HIV-1 Vpr is most likely produced in astrocytes where limited viral activity is stimulated by inflammation [21], resulting in localized effects. Our model simulates focal Vpr production by astrocytes in the hippocampus to examine if the toxic effect is sufficient to damage neurons both physically and functionally using histological analyses and behavioral testing. Vpr-treated rats, in comparison to their GFP-treated counterparts, were subjected to a 5-day protocol that resulted in impaired novelty recognition. Interestingly, although the Vpr gene expression was detectable at the RNA level across the duration, Vpr protein was detectible only transiently, indicating that a short exposure produces a pathological phenotype. Rats in both groups were able to demonstrate working memory of the testing chamber as demonstrated by reduction in exploration across three consecutive sessions to learn the object locations. Failure to learn the objects in this manner would constitute a short-term learning deficit. During acclimation to the open field, the rats construct a cognitive map of the objects and the environment that allows them to detect changes. If they are impaired they are unable to associate the spatial changes in the learning cage [64]. Interestingly, another study where Vpr was expressed transgenically in monocyte cells showed neurotoxic effects [28]. Vpr expression, in brain tissue, particularly in basal ganglia and cortex, was detected at the RNA level but as with our experiments, Vpr protein immunoreactivity was difficult to document [28]. In our model, Vpr treated rats show an overall reduction in distance (Figure 2A). In addition to distance traveled, we also measured time spent in center zone. Avoidance of the center zone indicates anxiety during the exposure to the open field [65]. Time in center zone was measured to assess Vpr’s effect on anxiety. We found minimal differences between the groups allowing us to exclude anxiety as a cause of the impaired memory (Figure 2B). Finally, as we reported in a similar study using Nef as the neurotoxin [41], we found that Vpr treatment does not affect general health as measured by weight gain (Figure 2C).

Our results correlate memory loss with neuronal chromatolysis in hippocampal CA3 region. Chromatolysis is typically seen in response to axonal injury and refers to the dissolution of Nissl bodies in the cytoplasm [66]. It is considered an early stage of morphological pathology that occurs in a cell that may enter apoptosis. Chromatolytic morphological changes have not been well explained, and there is a controversy as to whether chromatolytic neurons are irreversibly destined for death or if neurons may recover from injury marked by chromatolysis [52,53,67]. Chromatolysis is often observed as a consequence of axonal injury [68,69]. Some studies report that a cell undergoing chromatolysis activates regenerative pathways later, increasing protein production to counteract neuronal insults [70]. Redirecting cell machinery for regeneration can compromise metabolic needs and productivity that would otherwise be available for learning.

The hippocampus is involved in object recognition and memory formation [71]. To investigate the molecular mechanisms involved in HIV-1 Vpr induced memory impairment, we examined hippocampal levels of synaptophysin. Memory impairment was associated with decreased hippocampal synaptophysin in a mice model of Alzheimer [72]. As well, transgenic mice without synaptophysin demonstrate spatial and novelty learning impairments. Interestingly, these mice also demonstrate increased exploratory activity that is indicative of failure to habituate. Our Vpr-treated rats were able to habituate effectively, but like the transgenic mice lacking synaptophysin, demonstrated novelty learning impairment [54]. A major caveat to comparing these studies is that the transgenic mice had a complete absence of synaptophysin in all neurons and throughout development, whereas our Vpr rats showed decreased synaptophysin staining in a localized region for a short duration. This may explain why our rats showed habituation.

The CA3 is known to be associated with spatial learning, novelty detection, and short-term memory [73]. It is divided into CA3-a, −b, and -c subareas [74] CA3-a and -b, play important roles in new spatial information and short term memory [75]. As demonstrated in our results, the CA3-a region showed decreased synaptophysin staining that correlated with the learning and memory impairment found in the novel recognition spatial learning task. Synaptophysin plays an important role in modulating synaptic plasticity. Alterations to the metabolic needs of the cell may compromise consolidation process leading to a learning and memory impairment. Synaptophysin is a synaptic vesicle specific protein, present in the membrane of neuronal presynaptic vesicles [76,77]. It is implicated in the control of exocytosis [78] and neurotransmitter release [79]. Studies have shown increased synaptophysin in enriched environments with improved spatial memory [80]. These results demonstrate that HIV-Vpr expression in the hippocampus induces a synaptic impact with marked reductions in presynaptic synaptophysin protein involved in neurotransmission and synaptic plasticity.

## Conclusions

We report for the first time that Vpr produced endogenously from astrocytes from a discrete region (right hemisphere dentate gyrus) and short duration (five days or fewer) causes neurotoxicity that is both morphologically and functionally relevant. The functional impairment is specific for spatial and episodic memory and does not represent a generalizable impairment as animals demonstrate proper motor function as well as the capacity for short term learning. The impairment manifests at least in part from neuronal injury and synaptic damage specifically in the hippocampus. The absence of apoptotic cells suggests that the damage caused by transient expression of Vpr is not permanent. In some respects, this is akin to recovery in function shown by some patients upon starting suppressive therapy. However, our model does not address the issue of the impact of multiple, sporadic (temporal and anatomical) insults that could be caused by focal viral neurotoxin production. These events when summed over time may be at the foundation for the observed continued progression of HAND in treated HIV-1 infected populations. Our results provide a basis for the importance of sub-replication viral activity which may occur from atypically infected cells, like astrocytes, in the continued neurological morbidities affecting those infected with HIV-1 in the era of suppressive antiretroviral therapy.

## Abbreviations

cART: combination antiretroviral therapy; CNS: central nervous system; CSF: cerebrospinal fluid; GFP: green fluorescent protein; HAND: HIV-associated neurocognitive disorders; Vpr: viral protein R.

## Competing interests

The authors declare that they have no competing interests.

## Author’s contribution

LT performed the experiments. LT and RJN designed the study, analyzed the data and wrote the manuscript. Both authors read and approved the final manuscript.

## References

[B1] Mocroft A Ledergerber B Katlama C Kirk O Reiss P d’Arminio MA Knysz B Dietrich M Phillips AN Lundgren J Decline in the AIDS and death rates in the EuroSIDA study: an observational study Lancet 2003 362 22 29 10.1016/S0140-6736(03)13802-0 12853195

[B2] Panos G Samonis G Alexiou VG Kavarnou GA Charatsis G Falagas ME Mortality and morbidity of HIV infected patients receiving HAART: a cohort study Curr HIV Res 2008 6 257 260 10.2174/157016208784324976 18473789

[B3] Giometto B An SF Groves M Scaravilli T Geddes JF Miller R Tavolato B Beckett AA Scaravilli F Accumulation of beta-amyloid precursor protein in HIV encephalitis: relationship with neuropsychological abnormalities Ann Neurol 1997 42 34 40 10.1002/ana.410420108 9225683

[B4] An SF Giometto B Groves M Miller RF Beckett AA Gray F Tavolato B Scaravilli F Axonal damage revealed by accumulation of beta-APP in HIV-positive individuals without AIDS J Neuropathol Exp Neurol 1997 56 1262 1268 10.1097/00005072-199711000-00011 9370237

[B5] McArthur J Smith B Neurologic complications and considerations in HIV-infected persons Curr Infect Dis Rep 2013 15 61 66 10.1007/s11908-012-0312-2 23307491 PMC4313541

[B6] Heaton RK Clifford DB Franklin DR Jr Woods SP Ake C Vaida F Ellis RJ Letendre SL Marcotte TD Atkinson JH Rivera-Mindt M Vigil OR Taylor MJ Collier AC Marra CM Gelman BB McArthur JC Morgello S Simpson DM McCutchan JA Abramson I Gamst A Fennema-Notestine C Jernigan TL Wong J Grant I CHARTER Group HIV-associated neurocognitive disorders persist in the era of potent antiretroviral therapy: CHARTER Study Neurology 2010 75 2087 2096 10.1212/WNL.0b013e318200d727 21135382 PMC2995535

[B7] Tozzi V Balestra P Bellagamba R Corpolongo A Salvatori MF Visco-Comandini U Vlassi C Giulianelli M Galgani S Antinori A Narciso P Persistence of neuropsychologic deficits despite long-term highly active antiretroviral therapy in patients with HIV-related neurocognitive impairment: prevalence and risk factors J Acquir Immune Defic Syndr 2007 45 174 182 10.1097/QAI.0b013e318042e1ee 17356465

[B8] Heaton R Franklin D Ellis R McCutchan J Letendre S LeBlanc S Corkran S Duarte N Clifford D Woods S Collier AC Marra CM Morgello S Mindt MR Taylor MJ Marcotte TD Atkinson JH Wolfson T Gelman BB McArthur JC Simpson DM Abramson I Gamst A Fennema-Notestine C Jernigan TL Wong J Grant I CHARTER Group; HNRC Group HIV-associated neurocognitive disorders before and during the era of combination antiretroviral therapy: differences in rates, nature, and predictors J Neurovirol 2011 17 3 16 10.1007/s13365-010-0006-1 21174240 PMC3032197

[B9] Trillo-Pazos G Diamanturos A Rislove L Menza T Chao W Belem P Sadiq S Morgello S Sharer L Volsky DJ Detection of HIV-1 DNA in microglia/macrophages, astrocytes and neurons isolated from brain tissue with HIV-1 encephalitis by laser capture microdissection Brain Pathol 2003 13 144 154 12744468 10.1111/j.1750-3639.2003.tb00014.x PMC8096041

[B10] Tornatore C Nath A Amemiya K Major EO Persistent human immunodeficiency virus type 1 infection in human fetal glial cells reactivated by T-cell factor(s) or by the cytokines tumor necrosis factor alpha and interleukin-1 beta J Virol 1991 65 6094 6100 1920627 10.1128/jvi.65.11.6094-6100.1991 PMC250285

[B11] Price RW Brew B Sidtis J Rosenblum M Scheck AC Cleary P The brain in AIDS: central nervous system HIV-1 infection and AIDS dementia complex Science 1988 239 586 592 10.1126/science.3277272 3277272

[B12] Messam CA Major EO Stages of restricted HIV-1 infection in astrocyte cultures derived from human fetal brain tissue J Neurovirol 2000 6 Suppl 1 S90 S94 10871771

[B13] He J Chen Y Farzan M Choe H Ohagen A Gartner S Busciglio J Yang X Hofmann W Newman W Mackay CR Sodroski J Gabuzda D CCR3 and CCR5 are co-receptors for HIV-1 infection of microglia Nature 1997 385 645 649 10.1038/385645a0 9024664

[B14] Ghorpade A Nukuna A Che M Haggerty S Persidsky Y Carter E Carhart L Shafer L Gendelman HE Human immunodeficiency virus neurotropism: an analysis of viral replication and cytopathicity for divergent strains in monocytes and microglia J Virol 1998 72 3340 3350 9525661 10.1128/jvi.72.4.3340-3350.1998 PMC109814

[B15] Thompson KA Cherry CL Bell JE McLean CA Brain cell reservoirs of latent virus in presymptomatic HIV-infected individuals Am J Pathol 2011 179 1623 1629 10.1016/j.ajpath.2011.06.039 21871429 PMC3181362

[B16] Churchill MJ Wesselingh SL Cowley D Pardo CA McArthur JC Brew BJ Gorry PR Extensive astrocyte infection is prominent in human immunodeficiency virus–associated dementia Ann Neurol 2009 66 253 258 10.1002/ana.21697 19743454

[B17] Tornatore C Chandra R Berger JR Major EO HIV-1 infection of subcortical astrocytes in the pediatric central nervous system Neurology 1994 44 481 487 10.1212/WNL.44.3_Part_1.481 8145919

[B18] Ranki A Nyberg M Ovod V Haltia M Elovaara I Raininko R Haapasalo H Krohn K Abundant expression of HIV Nef and Rev proteins in brain astrocytes in vivo is associated with dementia AIDS 1995 9 1001 1008 10.1097/00002030-199509000-00004 8527071

[B19] Brack-Werner R Kleinschmidt A Ludvigsen A Mellert W Neumann M Herrmann R Khim MC Burny A Muller-Lantzsch N Stavrou D Infection of human brain cells by HIV-1: restricted virus production in chronically infected human glial cell lines AIDS 1992 6 273 285 10.1097/00002030-199203000-00004 1373627

[B20] Vincendeau M Kramer S Hadian K Rothenaigner I Bell J Hauck SM Bickel C Nagel D Kremmer E Werner T Leib-Mösch C Brack-Werner R Control of HIV replication in astrocytes by a family of highly conserved host proteins with a common Rev-interacting domain (Risp) AIDS 2010 24 2433 2442 10.1097/QAD.0b013e32833e8758 20827171

[B21] Li W Henderson LJ Major EO Al-Harthi L IFN-gamma mediates enhancement of HIV replication in astrocytes by inducing an antagonist of the beta-catenin pathway (DKK1) in a STAT 3-dependent manner J Immunol 2011 186 6771 6778 10.4049/jimmunol.1100099 21562161 PMC3167069

[B22] Tornatore C Meyers K Atwood W Conant K Major E Temporal patterns of human immunodeficiency virus type 1 transcripts in human fetal astrocytes J Virol 1994 68 93 102 8254781 10.1128/jvi.68.1.93-102.1994 PMC236268

[B23] Halassa MM Haydon PG Integrated brain circuits: astrocytic networks modulate neuronal activity and behavior Annu Rev Physiol 2010 72 335 355 10.1146/annurev-physiol-021909-135843 20148679 PMC3117429

[B24] Perea G Navarrete M Araque A Tripartite synapses: astrocytes process and control synaptic information Trends Neurosci 2009 32 421 431 10.1016/j.tins.2009.05.001 19615761

[B25] Navarrete M Perea G Fernandez de Sevilla D Gomez-Gonzalo M Nunez A Martin ED Araque A Astrocytes mediate in vivo cholinergic-induced synaptic plasticity PLoS Biol 2012 10 e1001259 10.1371/journal.pbio.1001259 22347811 PMC3279365

[B26] Deshpande M Zheng J Borgmann K Persidsky R Wu L Schellpeper C Ghorpade A Role of activated astrocytes in neuronal damage: potential links to HIV-1-associated dementia Neurotox Res 2005 7 183 192 10.1007/BF03036448 15897153

[B27] He J Choe S Walker R Di MP Morgan DO Landau NR Human immunodeficiency virus type 1 viral protein R (Vpr) arrests cells in the G2 phase of the cell cycle by inhibiting p34cdc2 activity J Virol 1995 69 6705 6711 7474080 10.1128/jvi.69.11.6705-6711.1995 PMC189580

[B28] Jones GJ Barsby NL Cohen EA Holden J Harris K Dickie P Jhamandas J Power C HIV-1 Vpr Causes Neuronal Apoptosis and In Vivo Neurodegeneration J Neurosci 2007 27 3703 3711 10.1523/JNEUROSCI.5522-06.2007 17409234 PMC6672409

[B29] Patel CA Mukhtar M Pomerantz RJ Human immunodeficiency virus type 1 Vpr induces apoptosis in human neuronal cells J Virol 2000 74 9717 9726 10.1128/JVI.74.20.9717-9726.2000 11000244 PMC112404

[B30] Piller SC Jans P Gage PW Jans DA Extracellular HIV-1 virus protein R causes a large inward current and cell death in cultured hippocampal neurons: implications for AIDS pathology Proc Natl Acad Sci USA 1998 95 4595 4600 10.1073/pnas.95.8.4595 9539783 PMC22535

[B31] Kitayama H Miura Y Ando Y Hoshino S Ishizaka Y Koyanagi Y Human immunodeficiency virus type 1 Vpr inhibits axonal outgrowth through induction of mitochondrial dysfunction J Virol 2008 82 2528 2542 10.1128/JVI.02094-07 18094160 PMC2258941

[B32] Rom I Deshmane SL Mukerjee R Khalili K Amini S Sawaya BE HIV-1 Vpr deregulates calcium secretion in neural cells Brain Res 2009 1275 81 86 19328187 10.1016/j.brainres.2009.03.024 PMC2692350

[B33] Levy DN Refaeli Y MacGregor RR Weiner DB Serum Vpr regulates productive infection and latency of human immunodeficiency virus type 1 Proc Natl Acad Sci U S A 1994 91 10873 10877 10.1073/pnas.91.23.10873 7971975 PMC45128

[B34] Pomerantz RJ Effects of HIV-1 Vpr on neuroinvasion and neuropathogenesis DNA Cell Biol 2004 23 227 238 10.1089/104454904773819815 15142380

[B35] Mukerjee R Chang JR Del Valle L Bagashev A Gayed MM Lyde RB Hawkins BJ Brailoiu E Cohen E Power C Azizi SA Gelman BB Sawaya BE Deregulation of microRNAs by HIV-1 Vpr Protein Leads to the Development of Neurocognitive Disorders J Biol Chem 2011 286 34976 34985 10.1074/jbc.M111.241547 21816823 PMC3186354

[B36] Gangwani MR Noel RJ Jr Shah A Rivera-Amill V Kumar A Human immunodeficiency virus type 1 viral protein R (Vpr) induces CCL5 expression in astrocytes via PI-3 K and MAPK signaling pathways J Neuroinflammation 2013 10 136 10.1186/1742-2094-10-136 24225433 PMC3831867

[B37] Ferrucci A Nonnemacher MR Wigdahl B Extracellular HIV-1 viral protein R affects astrocytic glyceraldehyde 3-phosphate dehydrogenase activity and neuronal survival J Neurovirol 2013 19 239 253 10.1007/s13365-013-0170-1 23728617 PMC3709860

[B38] Amini S Saunders M Kelley K Khalili K Sawaya BE Interplay between HIV-1 Vpr and Sp1 Modulates p21WAF1 Gene Expression in Human Astrocytes J Biol Chem 2004 279 46046 46056 10.1074/jbc.M403792200 15302882

[B39] Chauhan A Turchan J Pocernich C Bruce-Keller A Roth S Butterfield DA Major EO Nath A Intracellular human immunodeficiency virus Tat expression in astrocytes promotes astrocyte survival but induces potent neurotoxicity at distant sites via axonal transport J Biol Chem 2003 278 13512 13519 10.1074/jbc.M209381200 12551932

[B40] Bruce-Keller AJ Chauhan A Dimayuga FO Gee J Keller JN Nath A Synaptic Transport of Human Immunodeficiency Virus-Tat Protein Causes Neurotoxicity and Gliosis in Rat Brain J Neurosci 2003 23 8417 8422 12968004 10.1523/JNEUROSCI.23-23-08417.2003 PMC6740701

[B41] Chompre G Cruz E Maldonado L Rivera-Amill V Porter JT Noel RJ Jr Astrocytic expression of HIV-1 Nef impairs spatial and recognition memory Neurobiol Dis 2013 49 128 136 10.1016/j.nbd.2012.08.007 PMC3530662 22926191

[B42] Frangakis MV Kimelberg HK Dissociation of neonatal rat brain by dispase for preparation of primary astrocyte cultures Neurochem Res 1984 9 1689 1698 10.1007/BF00968079 6397695

[B43] Benice TS Rizk A Kohama S Pfankuch T Raber J Sex-differences in age-related cognitive decline in C57BL/6 J mice associated with increased brain microtubule-associated protein 2 and synaptophysin immunoreactivity Neuroscience 2006 137 413 423 10.1016/j.neuroscience.2005.08.029 16330151

[B44] Save E Poucet B Foreman N Buhot MC Object exploration and reactions to spatial and nonspatial changes in hooded rats following damage to parietal cortex or hippocampal formation Behav Neurosci 1992 106 447 456 1616611

[B45] Poucet B Spatial cognitive maps in animals: new hypotheses on their structure and neural mechanisms Psychol Rev 1993 100 163 182 8483980 10.1037/0033-295x.100.2.163

[B46] Moon HS Yang JS Role of HIV Vpr as a regulator of apoptosis and an effector on bystander cells Mol Cells 2006 21 7 20 16511342

[B47] Borjabad A Brooks AI Volsky DJ Gene expression profiles of HIV-1-infected glia and brain: toward better understanding of the role of astrocytes in HIV-1-associated neurocognitive disorders J Neuroimmune Pharmacol 2010 5 44 62 10.1007/s11481-009-9167-1 19697136 PMC3107560

[B48] Snyder A Ross MJ Murine models of Vpr-mediated pathogenesis Curr HIV Res 2009 7 129 135 10.2174/157016209787581526 19275581

[B49] Majno G Joris I Apoptosis, oncosis, and necrosis: an overview of cell death Am J Pathol 1995 146 3 15 7856735 PMC1870771

[B50] Barr ML Hamilton JD A quantitative study of certain morphological changes in spinal motor neurons during axon reaction J Comp Neurol 1948 89 93 121 10.1002/cne.900890203 18889710

[B51] Cammermeyer J Peripheral Chromatolysis after Transection of Mouse Facial Nerve Acta Neuropathol 1963 3 213 230 14190987 10.1007/BF00686415

[B52] Popratiloff A Kharazia VN Weinberg RJ Laonipon B Rustioni A Glutamate receptors in spinal motoneurons after sciatic nerve transection Neuroscience 1996 74 953 958 10.1016/0306-4522(96)00300-4 8895864

[B53] Hanz S Fainzilber M Retrograde signaling in injured nerve–the axon reaction revisited J Neurochem 2006 99 13 19 10.1111/j.1471-4159.2006.04089.x 16899067

[B54] Schmitt U Tanimoto N Seeliger M Schaeffel F Leube RE Detection of behavioral alterations and learning deficits in mice lacking synaptophysin Neuroscience 2009 162 234 243 10.1016/j.neuroscience.2009.04.046 19393300

[B55] Masliah E Achim CL Ge N DeTeresa R Terry RD Wiley CA Spectrum of human immunodeficiency virus-associated neocortical damage Ann Neurol 1992 32 321 329 10.1002/ana.410320304 1416802

[B56] Liu Y Liu H Kim BO Gattone VH Li J Nath A Blum J He JJ CD4-independent infection of astrocytes by human immunodeficiency virus type 1: requirement for the human mannose receptor J Virol 2004 78 4120 4133 10.1128/JVI.78.8.4120-4133.2004 15047828 PMC374297

[B57] Lopez-Herrera A Liu Y Rugeles MT He JJ HIV-1 interaction with human mannose receptor (hMR) induces production of matrix metalloproteinase 2 (MMP-2) through hMR-mediated intracellular signaling in astrocytes Biochim Biophys Acta 2005 1741 55 64 10.1016/j.bbadis.2004.12.001 15955449

[B58] Fields RD Stevens-Graham B New insights into neuron-glia communication Science 2002 298 556 562 10.1126/science.298.5593.556 12386325 PMC1226318

[B59] Barker AJ Ullian EM Astrocytes and synaptic plasticity Neuroscientist 2010 16 40 50 10.1177/1073858409339215 20236948

[B60] Atluri VS Kanthikeel SP Reddy PV Yndart A Nair MP Human synaptic plasticity gene expression profile and dendritic spine density changes in HIV-infected human CNS cells: role in HIV-associated neurocognitive disorders (HAND) PLoS One 2013 8 e61399 10.1371/journal.pone.0061399 23620748 PMC3631205

[B61] Ota Y Zanetti AT Hallock RM The role of astrocytes in the regulation of synaptic plasticity and memory formation Neural Plast 2013 2013 185463 24369508 10.1155/2013/185463 PMC3867861

[B62] Witcher MR Kirov SA Harris KM Plasticity of perisynaptic astroglia during synaptogenesis in the mature rat hippocampus Glia 2007 55 13 23 10.1002/glia.20415 17001633

[B63] Langford D Marquie-Beck J Almeida S Lazzaretto D Letendre S Grant I McCutchan JA Masliah E Ellis R Relationship of antiretroviral treatment to postmortem brain tissue viral load in human immunodeficiency virus-infected patients J Neurovirol 2006 12 100 107 10.1080/13550280600713932 16798671

[B64] Rudy JW Sutherland RJ The hippocampal formation is necessary for rats to learn and remember configural discriminations Behav Brain Res 1989 34 97 109 10.1016/S0166-4328(89)80093-2 2765175

[B65] Prut L Belzung C The open field as a paradigm to measure the effects of drugs on anxiety-like behaviors: a review Eur J Pharmacol 2003 463 3 33 10.1016/S0014-2999(03)01272-X 12600700

[B66] Clarke PG Clarke S Nineteenth century research on cell death Exp Oncol 2012 34 139 145 23069997

[B67] Glucksmann A Cell deaths in normal vertebrate ontogeny Biol Rev 1951 26 59 86 10.1111/j.1469-185X.1951.tb00774.x 24540363

[B68] Cragg BG What is the signal for chromatolysis? Brain Res 1970 23 1 21 10.1016/0006-8993(70)90345-8 4919474

[B69] Lieberman AR The axon reaction: a review of the principal features of perikaryal responses to axon injury Int Rev Neurobiol 1971 14 49 124 4948651 10.1016/s0074-7742(08)60183-x

[B70] Burnett MG Zager EL Pathophysiology of peripheral nerve injury: a brief review Neurosurg Focus 2004 16 E1 15174821 10.3171/foc.2004.16.5.2

[B71] Clarke JR Cammarota M Gruart A Izquierdo I Delgado-Garcia JM Plastic modifications induced by object recognition memory processing Proc Natl Acad Sci USA 2010 107 2652 2657 10.1073/pnas.0915059107 20133798 PMC2823877

[B72] Figueiredo CP Clarke JR Ledo JH Ribeiro FC Costa CV Melo HM Mota-Sales AP Saraiva LM Klein WL Sebollela A De Felice FG Ferreira ST Memantine rescues transient cognitive impairment caused by high-molecular-weight abeta oligomers but not the persistent impairment induced by low-molecular-weight oligomers J Neurosci 2013 33 9626 9634 10.1523/JNEUROSCI.0482-13.2013 23739959 PMC6619709

[B73] Kesner RP Lee I Gilbert P A behavioral assessment of hippocampal function based on a subregional analysis Rev Neurosci 2004 15 333 351 15575490 10.1515/revneuro.2004.15.5.333

[B74] Li XG Somogyi P Ylinen A Buzsaki G The hippocampal CA3 network: an in vivo intracellular labeling study J Comp Neurol 1994 339 181 208 10.1002/cne.903390204 8300905

[B75] Kesner RP Behavioral functions of the CA3 subregion of the hippocampus Learn Mem 2007 14 771 781 10.1101/lm.688207 18007020

[B76] Jahn R Schiebler W Ouimet C Greengard P A 38,000-dalton membrane protein (p38) present in synaptic vesicles Proc Natl Acad Sci USA 1985 82 4137 4141 10.1073/pnas.82.12.4137 3923488 PMC397950

[B77] Wiedenmann B Franke WW Identification and localization of synaptophysin, an integral membrane glycoprotein of Mr 38,000 characteristic of presynaptic vesicles Cell 1985 41 1017 1028 10.1016/S0092-8674(85)80082-9 3924408

[B78] Edelmann L Hanson PI Chapman ER Jahn R Synaptobrevin binding to synaptophysin: a potential mechanism for controlling the exocytotic fusion machine EMBO J 1995 14 224 231 7835333 10.1002/j.1460-2075.1995.tb06995.x PMC398074

[B79] Alder J Xie ZP Valtorta F Greengard P Poo M Antibodies to synaptophysin interfere with transmitter secretion at neuromuscular synapses Neuron 1992 9 759 768 10.1016/0896-6273(92)90038-F 1356373

[B80] Frick KM Fernandez SM Enrichment enhances spatial memory and increases synaptophysin levels in aged female mice Neurobiol Aging 2003 24 615 626 10.1016/S0197-4580(02)00138-0 12714119

